# Supplementary material for: Identification of Inflammation Markers as Novel Potential Predictors of the HIV-DNA Reservoir Size
Source: Int J Mol Sci. 2025 Aug 29;26(17):8430. doi: 10.3390/ijms26178430 (PMC12428898; doi:10.3390/ijms26178430)
Supplement: Supplementary file 1 [file ijms-26-08430-s001.zip › ijms-3789331-supplementary.pdf]

# Supplementary material

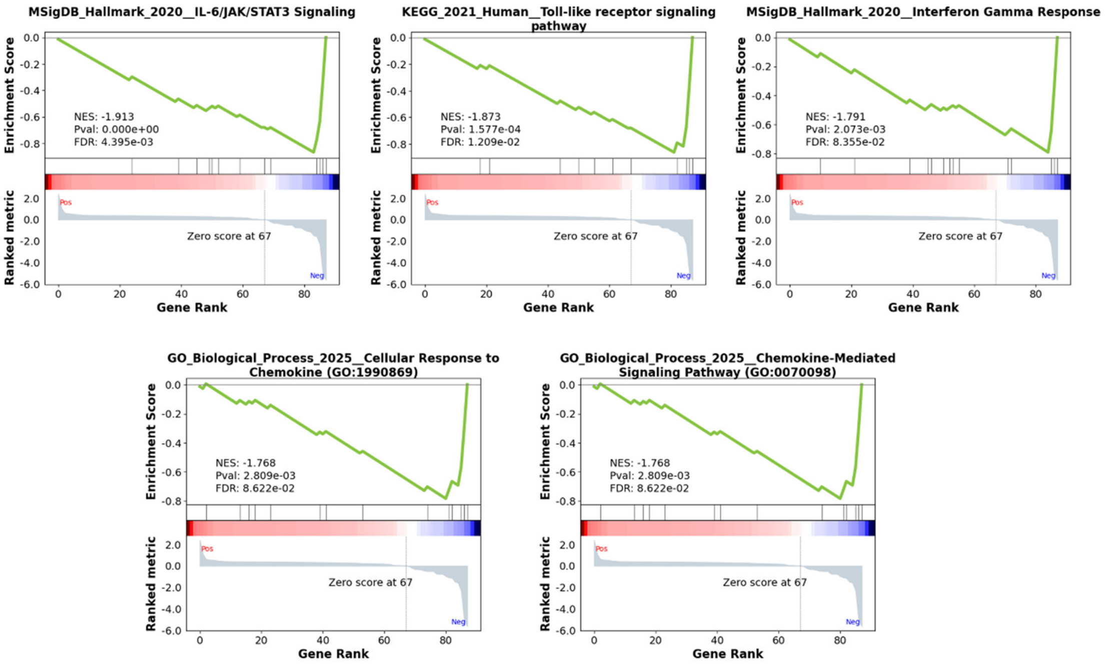


**Supplementary Figure 1.** Functional analysis of the differentially expressed markers among pre-ART and post-ART.

GSEA preranked approach was performed using the Python package gseapy (v1.1.6). The ranked protein list was analyzed against three gene set databases mentioned above. The significant gene sets were selected based on an *q*-value<0.15 (False Discovery Rate), and enrichment plots were generated for visualization of the significant results.

**Table S1.** Variation of inflammatory markers levels over ART administration.

| | Levels of markers [a] | | Fold-change [b] | Statistics [c] | |
|---|---|---|---|---|---|
| | Pre-ART | Post-ART | | *p*-value | *q*-value |
| **CXCL11** | 11.70 | 9.83 | 3.7 | 0.0001 | 0.0042 |
| **CXCL9** | 10.82 | 8.57 | 4.8 | 0.0001 | 0.0042 |
| **CXCL10** | 11.13 | 9.68 | 2.7 | 0.0005 | 0.014 |
| **CDCP1** | 5.33 | 4.03 | 2.5 | 0.002 | 0.028 |
| **SCF** | 8.55 | 9.17 | 1.5 | 0.0015 | 0.028 |
| **IL-18R1** | 9.58 | 9.28 | 1.2 | 0.0017 | 0.028 |
| **CCL19** | 12.19 | 11.17 | 2.0 | 0.0043 | 0.0452 |
| **CCL3** | 8.06 | 7.49 | 1.5 | 0.004 | 0.0452 |
| **IFN-gamma** | 10.04 | 8.40 | 3.1 | 0.0057 | 0.0532 |
| **IL18** | 11.23 | 10.04 | 2.3 | 0.0096 | 0.0672 |
| **TNFSF14** | 7.24 | 7.05 | 1.1 | 0.009 | 0.0672 |
| **TNFRSF9** | 8.02 | 7.36 | 1.6 | 0.0092 | 0.0672 |
| **CD8A** | 8.45 | 8.21 | 1.2 | 0.0175 | 0.1002 |
| **MCP-2** | 9.21 | 8.49 | 1.6 | 0.0179 | 0.1002 |
| **ADA** | 7.60 | 7.28 | 1.2 | 0.0176 | 0.1002 |
| **CD244** | 7.58 | 7.27 | 1.2 | 0.0201 | 0.1055 |
| **TRAIL** | 8.44 | 8.05 | 1.3 | 0.025 | 0.1222 |
| **PD-L1** | 7.96 | 7.34 | 1.5 | 0.0289 | 0.1222 |
| **IL10** | 3.69 | 2.76 | 1.9 | 0.0291 | 0.1222 |
| **DNER** | 8.44 | 8.65 | 1.2 | 0.0271 | 0.1222 |

[a] Levels of inflammatory markers are represented as median (log2).
[b] Fold-changes for each inflammatory marker were calculated as $2^{\text{(median pre-ART – median post-ART)}}$. For instance. the variation for CXCL11 levels over ART initiation was 3.7-fold ($2^{(11.7\text{-}9.83)}$). Fold-changes represents the decline of the markers levels. except for SCF and DNER that represent an increase.
[c] Statistical analysis: paired t-test (*p*-value) and false discovery rate (*q*-value) were employed.

**Table S2.** Levels of inf0lammatory markers associated with the HIV-DNA reservoir size at pre-ART.

| **Intact HIV-DNA** | | | | |
|---|---|---|---|---|
| **Marker** | **Estimate** | **Standard error** | **95% CI (asymptotic)** | ***p*-value** |
| **CX3CL1** | 0.6682 | 0.2901 | 0.05877 to 1.278 | 0.0334 |
| **ST1A1** | −0.2418 | 0.1076 | −0.4678 to −0.01575 | 0.0374 |
| **CD8A** | 1.153 | 0.5375 | 0.02372 to 2.282 | 0.0458 |
| **Defective HIV-DNA** | | | | |
| **Marker** | **Estimate** | **Standard error** | **95% CI (asymptotic)** | ***p*-value** |
| **MMP-10** | 0.8019 | 0.2374 | 0.3032 to 1.301 | 0.0034 |
| **ST1A1** | −0.2289 | 0.08325 | −0.4038 to −0.05399 | 0.0132 |
| **CXCL5** | −0.2431 | 0.1067 | −0.4674 to −0.01889 | 0.0352 |
| **AXIN1** | −0.2162 | 0.0957 | −0.4173 to −0.01516 | 0.0365 |
| **FGF-21** | 0.2111 | 0.09843 | 0.004282 to 0.4179 | 0.0459 |
| **Total HIV-DNA** | | | | |
| **Marker** | **Estimate** | **Standard error** | **95% CI (asymptotic)** | ***p*-value** |
| **MMP-10** | 0.6757 | 0.1797 | 0.2982 to 1.053 | 0.0014 |
| **ST1A1** | −0.1836 | 0.06547 | −0.3211 to −0.04604 | 0.0117 |
| **CXCL5** | −0.2219 | 0.08032 | −0.3906 to −0.05315 | 0.0128 |
| **GDNF** | 0.6098 | 0.2664 | 0.05000 to 1.170 | 0.0344 |
| **FGF-21** | 0.1705 | 0.07745 | 0.007783 to 0.3332 | 0.0410 |

Statistical analysis was performed using multivariate linear regression models adjusted by baseline (nadir) CD4 counts and inflammation markers levels (at pre-ART). HIV-DNA reservoir (Log10) at pre-ART was considered as the dependent variable and the other factors as the independent variables. A total of 21 individuals were analyzed, due to exclusion of two samples for multiple markers outliers.

**Table S3.** Levels of inflammatory markers associated with the HIV-DNA reservoir size at post-ART.

| **Intact HIV-DNA** | | | | |
|---|---|---|---|---|
| **Marker** | **Estimate** | **Standard error** | **95% CI (asymptotic)** | ***p*-value** |
| **IL-10** | −0.4262 | 0.1602 | −0.7615 to −0.09097 | 0.0154 |
| **Defective HIV-DNA** | | | | |
| **Marker** | **Estimate** | **Standard error** | **95% CI (asymptotic)** | ***p*-value** |
| **CXCL9** | −0.2746 | 0.1258 | −0.5354 to −0.01372 | 0.0400 |
| **TNF** | −0.411 | 0.1946 | −0.8183 to -0.003762 | 0.0481 |

Statistical analysis was performed using multivariate linear regression models adjusted by CD4 counts and inflammation markers levels (at post-ART). HIV-DNA reservoir (Log10) at post-ART was considered as the dependent variable and the other factors as the independent variables. A total of 22 individuals were analyzed. due to exclusion of one sample for multiple markers outliers.

**Table S4.** Predictor markers of the HIV-DNA reservoir decline

| **Intact HIV-DNA** | | | | |
|---|---|---|---|---|
| **Marker** | **Estimate** | **Standard error** | **95% CI (asymptotic)** | ***p*-value** |
| **SCF** | 0.9781 | 0.2234 | 0.5069 to 1.449 | 0.0004 |
| **CDCP1** | −0.3998 | 0.1519 | −0.7203 to −0.07931 | 0.0175 |
| **MCP-4** | −0.4854 | 0.2075 | −0.9233 to −0.04755 | 0.0318 |
| **ARTN** | −0.906 | 0.4115 | −1.774 to −0.03775 | 0.0418 |
| **IL-8** | −0.4405 | 0.2015 | −0.8657 to −0.01530 | 0.0431 |
| **Total HIV-DNA** | | | | |
| **Marker** | **Estimate** | **Standard error** | **95% CI (asymptotic)** | ***p*-value** |
| **MMP-10** | 0.6684 | 0.2771 | 0.08371 to 1.253 | 0.0275 |
| **Defective HIV-DNA** | | | | |
| **Marker** | **Estimate** | **Standard error** | **95% CI (asymptotic)** | ***p*-value** |
| **MMP-10** | 0.6021 | 0.2852 | 0.0003188 to 1.204 | 0.0499 |

Statistical analysis was performed using multivariate linear regression models adjusted by CD4 counts and inflammation markers levels (both at pre-ART). HIV-DNA reservoir (Log10) variation (difference between pre-ART – post-ART time points) was considered as the dependent variable and the other factors as the independent predictor variables.

**Table S5.** Dynamics of inflammatory markers associated with the intact HIV-DNA decline.

| Markers | Estimate | Standard error | 95% CI (asymptotic) | *p*-value |
|---|---|---|---|---|
| **SCF** | 0.6686 | 0.2329 | 0.1771 to 1.160 | 0.0106 |
| **IL-24** | −0.9649 | 0.3506 | −1.705 to −0.2251 | 0.0136 |
| **TNFRSF9** | −0.4943 | 0.2013 | −0.9189 to −0.06965 | 0.0251 |
| **ARTN** | −1.176 | 0.4942 | −2.219 to −0.1331 | 0.0293 |
| **TRAIL** | −0.9939 | 0.4601 | −1.965 to −0.02314 | 0.0453 |

Statistical analysis was performed using multivariate linear regression models adjusted by baseline (nadir) CD4 counts and inflammation markers variation (difference between pre-ART – post-ART time points). HIV-DNA reservoir (log10) variation (difference between pre-ART – post-ART time points) was considered as the dependent variable and the other factors as the independent predictor variables.

**Table S6.** Inflammatory markers levels before ART initiation depending of clinical characteristics.

| | **CD4 counts** | | | **Viral load** | | | **AIDS diagnosis** | | |
|---|---|---|---|---|---|---|---|---|---|
| | **Low CD4** | **High CD4** | ***p*-value** | **Low VL** | **High VL** | ***p*-value** | **Yes** | **No** | ***p*-value** |
| **CD8** | 8.5<br>[7.9 - 8.6] | 8.4<br>[8.2 - 8.9] | 0.25 | 8.3<br>[8.2 – 8.9] | 8.5<br>[8.2 – 8.6] | 0.50 | 8.3<br>[8.1 – 8.7] | 8.5<br>[8.2 – 8.8] | 0.74 |
| **ST1A1A** | 9.5<br>[8.0 - 11.4] | 10.9<br>[8.9 - 11.2] | 0.49 | 10.8<br>[9.5 -11.3] | 9.5<br>[7.2 - 11.3] | 0.21 | 10.2<br>[8.4 – 11.0] | 10.9<br>[7.8 -11.4] | 0.98 |
| **CX3CL1** | 3.7<br>[3.0 - 4.5] | 3.1<br>[2.8 - 3.7] | 0.82 | 2.9<br>[2.7 – 4.4] | 3.5<br>[3.1 – 4.0] | 0.63 | 3.9<br>[3.1 – 6.2] | 3.1<br>[2.7 – 3.8] | **0.03** |
| **SCF** | 8.2<br>[7.7 - 8.7] | 8.9<br>[8.1 - 9.1] | 0.14 | 8.6<br>[8.0 – 9.1] | 8.5<br>[7.9 – 9.1] | 0.88 | 8.7<br>[8.0 – 9.3] | 8.5<br>[8.0 – 9.1] | 0.42 |

* Values are indicated as medians and IQR. CD4 counts: low (<250 cells/mm3) or high CD4 counts (≥ 250 cells/mm3); viral load (VL): low (<100,000 copies HIV-RNA) or high (≥ 100,000 copies HIV-RNA); AIDS diagnosis (Yes/No). Statistics: comparative analysis were realized with t-test.

**Table S7.** Functional analysis of the inflammatory markers associated with the intact HIV reservoir size before ART initiation

| Gene_set | Term | P-value | Adjusted P-value | Genes |
|---|---|---|---|---|
| GO_Biological_Process_2025 | Lymphocyte Mediated Immunity (GO:0002449) | 0.0326085134471577 | 0.104514466 | CD8A |
| GO_Biological_Process_2025 | Negative Regulation of Interleukin-1 Beta Production (GO:0032691) | 0.0326085134471577 | 0.104514466 | CX3CL1 |
| GO_Biological_Process_2025 | Antigen Receptor-Mediated Signaling Pathway (GO:0050851) | 0.0326085134471577 | 0.104514466 | CD8A |
| GO_Biological_Process_2025 | Negative Regulation of Macrophage Activation (GO:0043031) | 0.0326085134471577 | 0.104514466 | CX3CL1 |
| GO_Biological_Process_2025 | Regulation of Interleukin-1 Alpha Production (GO:0032650) | 0.0326085134471577 | 0.104514466 | CX3CL1 |
| GO_Biological_Process_2025 | T Cell Mediated Immunity (GO:0002456) | 0.0326085134471577 | 0.104514466 | CD8A |
| GO_Biological_Process_2025 | T Cell Receptor Signaling Pathway (GO:0050852) | 0.0326085134471577 | 0.104514466 | CD8A |
| GO_Biological_Process_2025 | Synapse Pruning (GO:0098883) | 0.0326085134471577 | 0.104514466 | CX3CL1 |
| GO_Biological_Process_2025 | 3'-Phosphoadenosine 5'-Phosphosulfate Metabolic Process (GO:0050427) | 0.0326085134471577 | 0.104514466 | SULT1A1 |
| GO_Biological_Process_2025 | Positive Regulation of Actin Filament Bundle Assembly (GO:0032233) | 0.0326085134471577 | 0.104514466 | CX3CL1 |
| GO_Biological_Process_2025 | Oxoacid Metabolic Process (GO:0043436) | 0.0326085134471577 | 0.104514466 | SULT1A1 |
| GO_Biological_Process_2025 | Catecholamine Catabolic Process (GO:0042424) | 0.0326085134471577 | 0.104514466 | SULT1A1 |
| GO_Biological_Process_2025 | Regulation of Actin Filament Bundle Assembly (GO:0032231) | 0.0326085134471577 | 0.104514466 | CX3CL1 |
| GO_Biological_Process_2025 | Cell Junction Disassembly (GO:0150146) | 0.0326085134471577 | 0.104514466 | CX3CL1 |
| GO_Biological_Process_2025 | Estrogen Metabolic Process (GO:0008210) | 0.0326085134471577 | 0.104514466 | SULT1A1 |
| GO_Biological_Process_2025 | Ethanol Catabolic Process (GO:0006068) | 0.0326085134471577 | 0.104514466 | SULT1A1 |
| GO_Biological_Process_2025 | Ethanol Metabolic Process (GO:0006067) | 0.0326085134471577 | 0.104514466 | SULT1A1 |
| GO_Biological_Process_2025 | Regulation of Neuron Migration (GO:2001222) | 0.0326085134471577 | 0.104514466 | CX3CL1 |
| GO_Biological_Process_2025 | Regulation of Neuron Projection Development (GO:0010975) | 0.0326085134471577 | 0.104514466 | CX3CL1 |
| GO_Biological_Process_2025 | Integrin Activation (GO:0033622) | 0.0326085134471577 | 0.104514466 | CX3CL1 |
| GO_Biological_Process_2025 | Positive Regulation of Cell Projection Organization (GO:0031346) | 0.0326085134471577 | 0.104514466 | CX3CL1 |
| GO_Biological_Process_2025 | Purine Ribonucleoside Bisphosphate Metabolic Process (GO:0034035) | 0.0326085134471577 | 0.104514466 | SULT1A1 |
| GO_Biological_Process_2025 | Neuron Maturation (GO:0042551) | 0.0326085134471577 | 0.104514466 | CX3CL1 |
| GO_Biological_Process_2025 | Negative Regulation of Microglial Cell Activation (GO:1903979) | 0.0326085134471577 | 0.104514466 | CX3CL1 |
| GO_Biological_Process_2025 | Dopamine Catabolic Process (GO:0042420) | 0.0326085134471577 | 0.104514466 | SULT1A1 |
| GO_Biological_Process_2025 | Purine Ribonucleotide Metabolic Process (GO:0009150) | 0.0326085134471577 | 0.104514466 | SULT1A1 |
| GO_Biological_Process_2025 | Dopamine Metabolic Process (GO:0042417) | 0.0326085134471577 | 0.104514466 | SULT1A1 |
| GO_Biological_Process_2025 | Neuron Remodeling (GO:0016322) | 0.0326085134471577 | 0.104514466 | CX3CL1 |
| GO_Biological_Process_2025 | Regulation of Cell Development (GO:0060284) | 0.0326085134471577 | 0.104514466 | CX3CL1 |
| GO_Biological_Process_2025 | Positive Regulation of Neuron Projection Development (GO:0010976) | 0.0326085134471577 | 0.104514466 | CX3CL1 |
| GO_Biological_Process_2025 | Negative Regulation of Neuron Migration (GO:2001223) | 0.0326085134471577 | 0.104514466 | CX3CL1 |
| GO_Biological_Process_2025 | Negative Regulation of Cell Adhesion (GO:0007162) | 0.0326085134471577 | 0.104514466 | CX3CL1 |
| GO_Biological_Process_2025 | Steroid Metabolic Process (GO:0008202) | 0.0326085134471577 | 0.104514466 | SULT1A1 |
| GO_Biological_Process_2025 | Regulation of Cell-Substrate Adhesion (GO:0010810) | 0.0326085134471577 | 0.104514466 | CX3CL1 |
| GO_Biological_Process_2025 | Primary Alcohol Catabolic Process (GO:0034310) | 0.0326085134471577 | 0.104514466 | SULT1A1 |
| GO_Biological_Process_2025 | Negative Regulation of Cell Motility (GO:2000146) | 0.0326085134471577 | 0.104514466 | CX3CL1 |
| GO_Biological_Process_2025 | Regulation of Lipopolysaccharide-Mediated Signaling Pathway (GO:00316 | 0.0326085134471577 | 0.104514466 | CX3CL1 |
| GO_Biological_Process_2025 | Negative Regulation of Cell-Substrate Adhesion (GO:0010812) | 0.0326085134471577 | 0.104514466 | CX3CL1 |
| GO_Biological_Process_2025 | Sulfation (GO:0051923) | 0.0326085134471577 | 0.104514466 | SULT1A1 |

The Enrichr API (https://maayanlab.cloud/Enrichr/) was used via the Python package gseapy (v1.1.6) to test gene sets using an Over-Representation Analysis (ORA). The protein list (converted to gene symbols) significantly associated with conditions of interest in previous analyses were tested against three gene set databases: GO_Biological_Process_2025. MSigDB_Hallmark_2020. and KEGG_2021_Human. Significant gene sets were selected based on an adjusted *p*-value < 0.15.
